# Soil and Soilless Tomato Cultivation Promote Different Microbial Communities That Provide New Models for Future Crop Interventions

**DOI:** 10.3390/ijms23158820

**Published:** 2022-08-08

**Authors:** Alice Anzalone, Alexandros Mosca, Giulio Dimaria, Daniele Nicotra, Matilde Tessitori, Grete Francesca Privitera, Alfredo Pulvirenti, Cherubino Leonardi, Vittoria Catara

**Affiliations:** 1 Department of Agriculture, Food and Environment, University of Catania, 95123 Catania, Italy; 2 Department of Physics and Astronomy, University of Catania, 95123 Catania, Italy; 3 Department of Clinical and Experimental Medicine, University of Catania, 95123 Catania, Italy

**Keywords:** tomato, bacterial community, fungal community, biocontrol agents, plant pathogens, tomato growing stages

## Abstract

The cultivation of soilless tomato in greenhouses has increased considerably, but little is known about the assembly of the root microbiome compared to plants grown in soil. To obtain such information, we constructed an assay in which we traced the bacterial and fungal communities by amplicon-based metagenomics during the cultivation chain from nursery to greenhouse. In the greenhouse, the plants were transplanted either into agricultural soil or into coconut fiber bags (soilless). At the phylum level, bacterial and fungal communities were primarily constituted in all microhabitats by Proteobacteria and Ascomycota, respectively. The results showed that the tomato rhizosphere microbiome was shaped by the substrate or soil in which the plants were grown. The microbiome was different particularly in terms of the bacterial communities. In agriculture, enrichment has been observed in putative biological control bacteria of the genera *Pseudomonas* and *Bacillus* and in potential phytopathogenic fungi. Overall, the study describes the different shaping of microbial communities in the two cultivation methods.

## 1. Introduction

Tomato, with 180 Mt of production and 5.03 Mha of cultivated area, is the second most important vegetable crop worldwide after potato and the most important vegetable crop for fruit [1]. Its high consumption and content of health-promoting compounds are considered a very important component of the modern diet [2]. Italy, Spain and Turkey are the largest producers in the Mediterranean region, in terms of both processed tomatoes and fresh tomatoes consumed.

Soilless cultivation in greenhouses has increased considerably due to, for example, the reduction in soil-borne diseases, and optimization of plant nutrition [3]. Many soilless growing systems are based on solid rooting media called ‘growing media’ or ‘substrates’ [4].

The choice of growing medium is a very important aspect in soilless cultivation. Materials differ greatly in terms of physical and chemical characteristics. Little information is available on the microbial composition of substrates and is mainly based on microbial culture. Substrates derived from sieved volcanic minerals heated to 700–1000 °C (e.g., rockwool, perlite) are virtually free of life [3], while other substrates have quite low microbial contamination (e.g., peat or coconut fiber), which increases rapidly by introducing the plant and irrigation water [5].

Although the control of soil-borne diseases is one of the most important reasons for the development of soilless culture, some associated diseases have been described in it [6]. Additionally, minor infections with opportunistic pathogens, sometimes not reported elsewhere, have been described. Tomato disease outbreaks exclusively for the soilless cultivations or more severe than in the soil have been described [6,7,8,9,10].

Microbial communities play a central role in plant health and productivity [11,12,13]. Their composition and structure fluctuate according to the organs and compartments of the plant (e.g., rhizosphere, ectorhizosphere, phyllosphere, and endosphere), which are specific habitats for microbial colonization [12,14,15,16,17]. The greatest attention has been given to the rhizosphere where there is a very active microbial interaction as the exudates released by plant roots are the main food source for microorganisms and a driving force for their population density and their activities [14,18]. These communities establish a dense network of neutral, pathogenic or beneficial interactions with plants [18,19]. A subset of them penetrate plant roots and become endophytes, residing asymptomatically in plant tissues (endosphere), without causing disease [20,21]. Alongside this horizontal transmission, derived from the soil environment, vertical transmission via seeds has also been demonstrated, although to a lesser extent [22,23,24,25].

The endophyte microbiome is complex and asymptomatic plant tissues harbor both beneficial (mutualistic), neutral (commensal) and potentially harmful (pathogenic) microorganisms [20,21,26]. The latter can include both plant and human pathogens that affect plant growth and health [18,27,28].

Metagenomics studies have also improved the understanding of tomato microbiome formation in the root environment. Studies have focused on the composition of microbial communities in the rhizosphere and endorhizosphere of tomato in different soils [29,30], tomato genotypes [31,32,33], rootstocks [34] as well as in relation to seed transmission [23], crop management [35,36,37], and soil-borne diseases [38,39,40,41].

It has been observed that the microbial communities of tomato in the rhizosphere and rhizoplane show the greatest richness and diversity compared to those in the endorhizosphere [30,42,43]. Soil structure and composition affect the microbial community of the rhizosphere and to a lesser extent the endorhizosphere [23,33]. Significant differences were also observed between native and agricultural soils and commercial substrates [44]. Chialva and co-authors (2018) [45] demonstrated that native soil components induce a state of alertness in the plant, shaping the molecular responses of tomato roots by enhancing gene induction involved in defensive responses. Vertical transmission has also been demonstrated, as by studying seed endophytic microbial communities over two generations, continuous turnover of bacterial and archaeal seed assemblage was observed [23,33]. Seeds also act as a carrier of beneficial bacteria over seed generations [23].

Despite more than ten years of research, there are no studies on the formation and evolution of the tomato microbiome under commercial conditions from seed to nursery plants for transplantation and then greenhouse cultivation. Multicomponent processes (i.e., biotic and abiotic) play an important role in determining the microbial communities of crops that influence plant productivity and health. The present study aimed to explore the evolution of the tomato root microbiome (rhizosphere and endorhizosphere) in soil and soilless tomatoes grown in greenhouses. For this purpose, seedlings grown on virgin substrates and/or containers were transplanted into common agricultural soil or above-ground containers with an inert substrate where the nutritional component was provided by nutrient solutions. The profound differences in microbial communities (bacteria and fungi) observed in the two culture systems are discussed in this study with particular attention to those with potential biological or pathogen control behavior.

## 2. Results

### 2.1. General Structure of the Tomato Root Associated Microbiome in the Seed, in Nursery and in Greenhouse in Soil and Soilless Cultivation Systems

**Figure 1:**
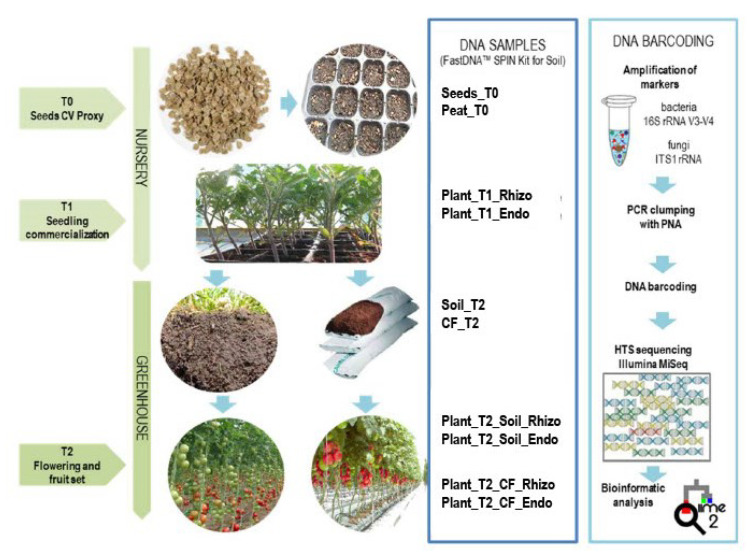
Experimental design workflow for sampling and processing to study bacterial and fungal communities by amplicon-based metagenomics.

Our study began with the sampling of tomato seeds (genotype) and a growth substrate (peat) in a commercial nursery and then again at the seedling marketing stage, one month after sowing. This is the period when seedling microbial communities may have been affected by the nursery environment and cropping systems, e.g., irrigation, and fertilization. The seedlings transplanted in the greenhouse, either in agricultural soil or in above-ground coconut fiber bags, were again sampled two months later. Bacterial and fungal communities of the seed endosphere, rhizo- and endorhizosphere of tomato seedlings in the nursery and plants in the greenhouse were analyzed, as well as all growing substrates (in the nursery and in the greenhouse) and agricultural soil (Figure 1 and Supplementary Table S1).

Illumina sequencing of bacterial 16S rRNA and fungal ITS yielded 2,190,541 and 4,936,217 sequences, respectively. After discarding chimeras, singletons, chloroplast and non-microbial reads, 583,882 bacterial and 3,719,440 fungal reads remained. The highest proportion of reads assigned to a plant taxonomic identification (up to 74%) was observed for 16S rRNA amplification in the endorhizosphere and seed samples as the plant tissues were crushed during extraction.

Bioinformatic reconstruction of the bacterial and fungal community identified a total of 7599 and 4886 distinct features, respectively. Microbial communities in tomato plants in the nursery and greenhouse under both growing conditions, i.e., agricultural soil and coconut fiber as well as communities in the seed, soil, substrates and in the different cultivation are shown in Supplementary Figure S1.

Bacterial communities were dominated by Proteobacteria in all microhabitats except the rhizosphere of plants grown in agricultural soil (Plant_T2_Soil_Rhizo) (Supplementary Figure S1A). Overall, in the endorhizosphere and rhizosphere of plants grown on substrates of coconut fiber (Plant_T2_CF) and peat (Plant_T1), Protebacteria represent 51, 52, 68, and 44% of the bacterial communities, respectively. The bacterial communities of the rhizosphere of plants grown in soil (Plant_T2_Soil_Rhizo) were represented by 30% Proteobacteria and Bacteroidetes (Supplementary Figure S1A).

In seeds (Seeds_T0), highly sequenced phyla included Proteobacteria (45%), Actinobacteria (29%), Firmicutes (12%), Bacteroidetes (9%) (Supplementary Figure S1A). Soil (Soil_T2), and growth substrates (Peat_T0 and CF_T2) showed a profoundly different composition (Supplementary Figure S1A). Representative phyla were Actinobacteria (up to 50% in coconut fiber substrate, CF_T2), Firmicutes and Bacteroidetes. Verrucomicrobia, Chloroflexi, and Planctomycetes were predominantly found in all samples, although at different concentrations (Supplementary Figure S1A).

Fungal communities were dominated by a few dominant phyla (Supplementary Figure S1B). Ascomycota was the most represented phylum in all samples. Fungal communities in the rhizosphere of plants grown in soil (Plant_T2_Soil_Rhizo) were characterized by a high percentage of Olpidiomycota (46%). This latter phylum was present in a small percentage in the other samples. The highest percentage of Basidiomycota was observed in the rhizosphere of plantlets grown in the nursery (Plant_T1_Rhizo) (22%). Of the other samples analyzed (seeds, soil, substrates), Mortierellomycota were enriched with agricultural soil (Soil_T2) (Supplementary Figure S1B).

#### 2.1.1. Taxonomic Distributions of Bacterial Communities

**Figure 2:**
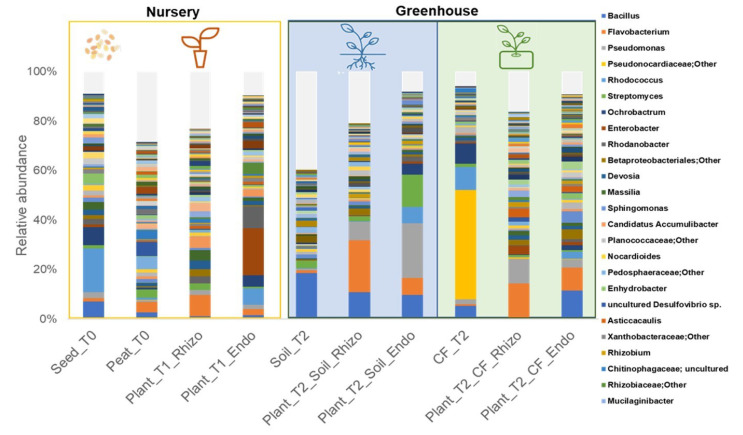
Distribution of bacterial communities in nursery samples, seeds (Seeds_T0), peat substrate (Peat_T0) and rhizo and endorhizosphere of plantlets (Plant_T1_Rhizo; Plant_T1_Endo); and greenhouse, coconut fiber substrate (CF_T2) and agricultural soil (Soil_T2), rhizo and endorhizosphere of tomato plants in the two cropping systems (Plant_T2_Soil_Rhizo; Plant_T2_CF_Rhizo, Plant_T2_Soil_Endo; Plant_T2_CF_Endo), at the genus level. Bar charts represent bacterial community composition (only key taxa have been included >1%). The legend refers to the first 25 bacteria depicted at the bottom of the bars.

The taxonomy of the bacterial community was examined at the genus level (Figure 2). All culture substrates (Peat_T0, CF_T2) were characterized by profound differences within their bacterial communities. Seeds (Seeds_T0), agricultural soil (Soil_T2) and coconut fiber substrate (CF_T2) dominant bacterial taxa were *Rhodococcus* (17.6%), *Bacillus* (18.6%) and Pseudonocardiaceae (44.2%), respectively. The most abundant genus in the bacterial communities of rhizosphere environments was the genus *Flavobacterium* (8.6, 20.9 and 13.6%, in Plant_T1_Rhizo; Plant_T2_Soil_Rhizo and Plant_T2_CF_Rhizo; respectively). Bacterial communities in the endorhizosphere of nursery-grown seedlings (Plant_T1_Endo) were dominated by the genus *Enterobacter.* The endorhizophere of plants grown in agricultural soil (Plant_T2_Soil_Endo) was dominated by the genera *Pseudomonas* (22.2%) and *Streptomyces* (13%) genera, while that of plants grown in the coconut fiber substrate (Plant_T2_CF_Endo) was characterized by genera *Bacillus* (11.3%) and *Flavobacterium* (9.3%). Compared to ectorhizosphere environments, the compositions of bacterial endophytes were quite different and their distributions also varied in the different growth substrates (Figure 2).

#### 2.1.2. Taxonomic Distribution of Fungal Communities

**Figure 3:**
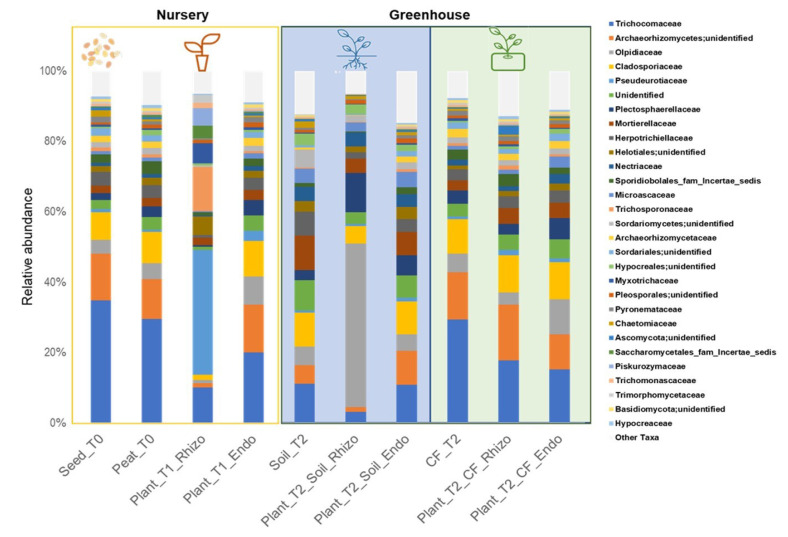
Distribution of fungal communities in nursery samples, seeds (Seeds_T0), peat substrate (Peat_T0) and rhizo and seedling endorhizosphere (Plant_T1_Rhizo; Plant_T1_Endo); and greenhouse, coconut fiber substrate (CF_T2) and agricultural soil (Soil_T2), rhizo and endorhizosphere of tomato plants in the two cultivation systems (Plant_T2_Soil_Rhizo; Plant_T2_CF_Rhizo, Plant_T2_Soil_Endo; Plant_T2_CF_Endo), bar charts represent fungal community composition (only key taxa have been included >1%).

Fungal community taxonomy was examined at the family level (Figure 3). The seeds (Seeds_T0) and the two growth substrates (Peat_T0 and CF_T2) were dominated by the Trichocomaceae family (approximately 30%). On the other hand, the agricultural soil (Soil_T2) showed a profoundly different composition.

The rhizosphere fungal communities in the nursery (Plant_T1_Rhizo) were characterized by a high abundance of the Pseudeurotiaceae family (35.5%). The rhizosphere of plants grown in the coconut fiber substrate (Plant_T2_CF_Rhizo) showed Archaeorhizomycetes, Cladosporiaceae, Mortierellaceae, as the most abundant taxa. In contrast, those of plants grown in agricultural soil (Plant_T2_Soil_Rhizo) were characterized by a lower abundance of Trichocomaceae and a very high abundance of Olpidiaceae (Figure 3 and Supplementary Figure S3).

The family Trichocomaceae in the endorhizosphere was the most abundant taxon in all cropping systems. In the nursery production materials (Peat_T0, and Seeds_T0), the most abundant fungal endophyte taxa were Trichocomaceae, Archaeorhizomycetes, Cladosporiaceae, and Olpidiaceae (Figure 3).

### 2.2. Analysis of Diversity Indices

#### 2.2.1. Richness and Diversity of Microbial Communities

**Figure 4:**
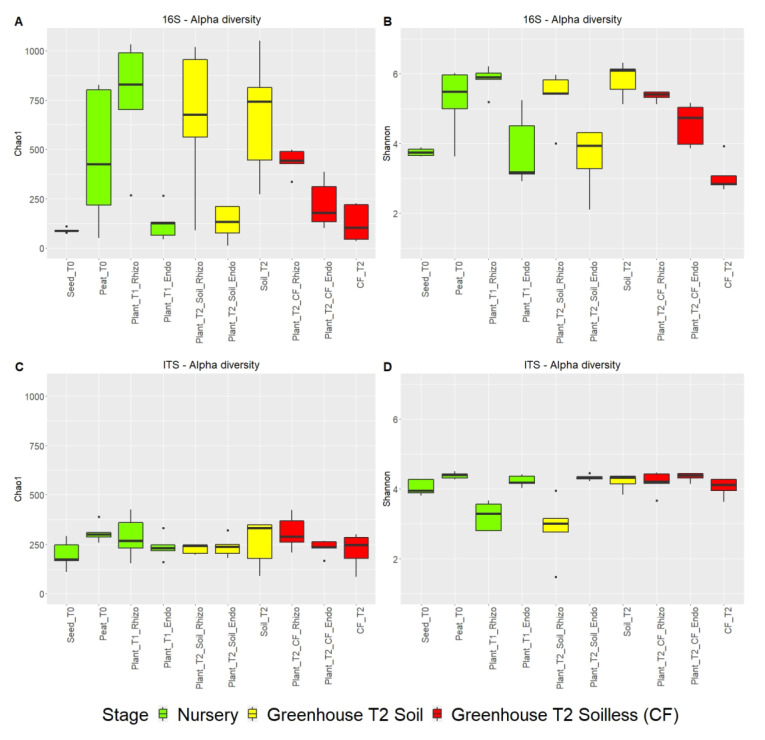
Estimation of the alpha diversity of the microbiome bacterial community (**A**,**B**); and fungal community (**C**,**D**) in the different samples of the tomato crop chain based on amplicon sequencing data. The observed Chao1 and Shannon indices were used in the alpha diversity analysis.

The microbial alpha diversity, estimated by Chao1 richness and Shannon diversity indices, was higher in the bacterial community than in the fungal community (Figure 4A–D and Supplementary Tables S2 and S3).

Based on the Chao1 index, the highest bacterial community richness was observed in the rhizospheres of nursery plantlets (Plant_T1) and peat (Peat_T0) and in plants grown in agricultural soil (Plant_T2_Soil) and the soil itself (Soil_T2). The bacterial richness in the rhizosphere of plants grown in coconut fiber (Plant_T2_CF_Rhizo) was significantly lower than that of the substrate before transplanting. Overall, the richness of the rhizosphere two months after transplanting into the coconut fiber substrate was significantly lower than that of the plants grown in the soil although they came from the same batch of nursery seedlings. The lowest richness of plant-associated bacterial communities was observed in endosphere environments (seeds and endorhizospheres) (Figure 4A). The Shannon index was analyzed to represent the diversity of bacterial species. The bacterial communities in the peat substrate (Peat_T0) and in the agricultural soil (Soil_T2) showed the greatest diversity as well as the rhizospheres of the plants grown herein (Figure 4B).

**Figure 5:**
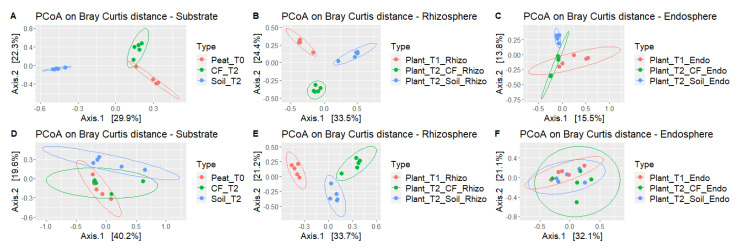
PCoA of bacterial (**A**–**C**); and fungal communities (**D**–**F**) based on amplicon sequencing data. Sample clustering was based on the Bray–Curtis dissimilarity matrix. Each point on the graph corresponds to a single sample (biological replication).

The fungal communities of the different sample groups did not show significant differences in richness (Figure 5C). Interestingly, fungal communities in the rhizosphere of plants grown in peat (Plant_T1_Rhizo) and in coconut fiber substrate (Plant_T2_CF_Rhizo) showed a significant reduction in diversity (Figure 4C,D and Supplementary Tables S2 and S3).

#### 2.2.2. Comparison of Microbial Communities

PCoA of beta diversity (Bray–Curtis dissimilarity) was performed to test the effects of the isolation source (seeds, soil, growth substrates, rhizosphere, and endorhizophere) as well as the various stages in the growth chain of tomato from the nursery to commercial greenhouse production on the microbial compositions (Figure 5A–F and Supplementary Figure S2).

Bacterial communities in growing media, peat in nursery (Peat_T0) and coconut fiber (CF_T2) and agricultural soil (Soil_T2) in the greenhouse showed clearly and significantly different signatures. Tomato rhizosphere bacterial communities changed drastically from the nursery to the commercial greenhouse during the flowering and fruiting phenological phase (i.e., two months after transplanting) both in the plants grown in agricultural soil (Plant_T2_Soil) and coconut fiber (Plant_T2_CF). Additionally, rhizospheres grown in agricultural soils and coconut fiber showed significant distances. Different signatures were also observed between bacterial communities in the endorhizosphere of plants grown in agricultural soil (Plant_T2_ Soil_Endo) and in coconut fiber (Plant_T2_CF_Endo) (Figure 5A–C). Beta diversity analysis showed that the fungal communities of the agricultural soil (Soil_T2) differed from those of the two substrates (Peat_T0 and CF_T2). Fungal communities in the rhizosphere, but not in the endorhizosphere, of plants grown in agricultural soil (Plant_T2_Soil) and coconut fiber (Plant_T2_CF) differed significantly from each other and from communities observed in nursery plantlets (Plant_T1).

Beta diversity analysis highlighted the diversity of fungal communities, which showed a high dispersion in all samples except for the rhizosphere samples (Figure 5D–F).

Considering the beta diversity of the growth environments (Supplementary Figure S2), in nursery conditions, the bacterial communities of the endosphere environments (Seeds_T0 and Plant_T1_Endo) showed common signatures that differed from those of the substrate of peat (Peat_T0) and seedling rhizosphere (Plant_T1_Rhizo), while the bacterial communities under greenhouse conditions in the different compartments (soil, substrate, rhizosphere and endorhizosphere) were more clearly distinguished. This was not the case for the fungal communities in the nursery and greenhouse (Supplementary Figure S2).

#### 2.2.3. Tomatoes Grown in Soil and Soilless Media Attract Different Microbiome Communities

The evolution of the structure and composition of bacterial and fungal communities was studied at the genus level by differential abundance analysis.

Significant changes were observed between the microbial communities from seeds to plants, in the nursery, and then after transplantation in the greenhouse in the agricultural soil and in the substrate (Supplementary Tables S4–S17).

In particular, bacterial communities of plant rhizospheres grown in agricultural soil (Plant_T2_Soil_Rhizo) and in coconut fiber substrate (Plant_T2_CF_Rhizo) showed more bacteria enriched than depleted compared to those grown in peat (Plant_T1_Rhizo) (Supplementary Figure S3 and Supplementary Tables S4 and S5). The endospheres of the plants grown in the nursery (Plant_T1_Endo) showed three significantly enriched bacterial families (Cellvibrionaceae, Rhizobiaceae and Caulobacteriaceae) compared to the seeds (Seed_T0) (Supplementary Figure S4 and Supplementary Table S7) and the endospheres of the plants in the greenhouse (Plan_T2_Soil_Endo, Plant_T2_CF_Endo) were depleted compared to those in the nursery (Supplementary Figure S4 and Supplementary Tables S8 and S9).

Fungal communities only showed variations in the rhizospheres of the transplanted plants (Plant_T2_Soil_Rhizo), which showed less abundant taxa compared to the nursery plant (Plant_T1_Rhizo) (Supplementary Figures S5, S6 and Supplementary Table S12).

Since up to the time of transplantation the plants shared both genetic source and growing conditions, we focused more on comparing the differences between the soil and soilless cultivation, which was the key objective of our analysis.

**Figure 6:**
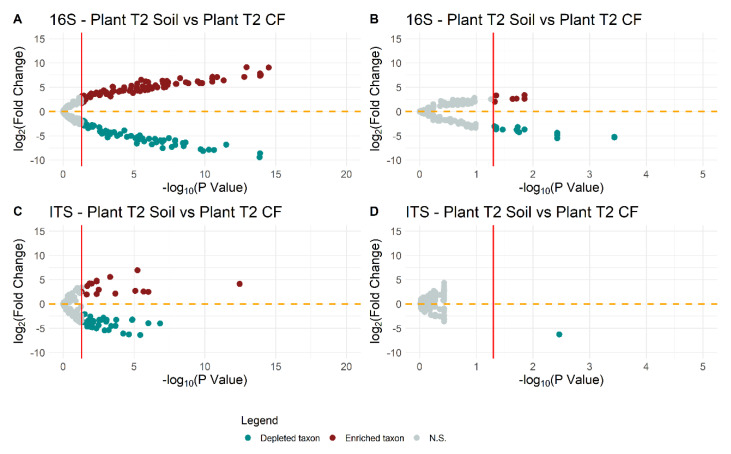
Volcano plots showing differentially abundant bacteria and fungi in rhizosphere (**A**,**C**) and endorhizosphere (**B**,**D**), respectively. Negative log-transformed false-discovery rate (FDR) P-values are plotted on the x-axis and log fold change is plotted on the y-axis. The orange dotted horizontal line represents the log fold change cut-off which divides the significant enriched bacteria and fungi (red dots) and the significant depleted bacteria and fungi (blue dots) from the non-significant (grey dots). The red vertical line represents the 1.30 negative cut-off log *p*-value (which corresponds to the FDR 0.05 cut-off).

There was a significant depletion and enrichment of bacterial and fungal taxa in the rhizosphere and endorhizosphere of plants grown in agricultural soil compared to those grown in coconut fiber (soilless) (Figure 6).

**Figure 7:**
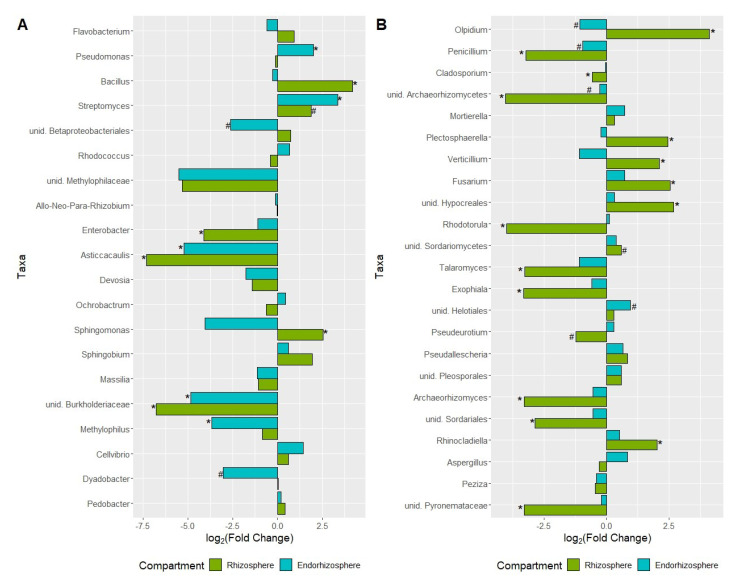
Differentially abundant taxa of the bacterial (**A**) and fungal (**B**) communities in the endorhizosphere and rhizosphere of tomatoes grown in agricultural soil versus those grown in soilless cultivation. The position along the x axis represents the fold change between the two different cultivations in greenhouse. All the tests were performed considering *p*-value (#) and the adjusted *p*-value with the false-discovery rate method (*, *p*-value FDR < 0.05).

Figure 7A,B show the differential analysis changes from the baseline [log fold change (logFC)], of the taxa with a relative abundance greater than 1% in the rhizosphere and endorhizosphere of plants under both conditions. The bacterial taxa enriched in the rhizosphere of plants grown in agricultural soil were: *Bacillus* (Firmicutes) (*p* < 0.005); *Flavobacterium* (Bacteroidetes); *Streptomyces* (Actinobacteria); *Sphingomonas* (*p* = 0.04) and *Sphingobium* (Proteobacteria) (Figure 7A).

On the other hand, *Pseudomonas* and *Cellvibrio* (Proteobacteria) and *Streptomyces* (Actinobacteria) were enriched in the endorhizosphere. The taxa depleted in the rhizosphere all belong to the phylum Proteobacteria and included: Methylophilaceae (family) (*p* < 0.001); *Enterobacter* (*p* < 0.01), *Asticcacaulis* (*p* < 0.001), *Devosia*, *Massilia*, Burkholderiaceae (family) (*p* < 0.001), and *Methylophilus*. On the other hand, Betaproteobacteriales (order), Methylophilaceae (family), *Enterobacter*, *Asticcacaulis* (*p* < 0.001), *Devosia, Massilia*, Burkholderiaceae (family) (*p* < 0.001), and *Methylophilus* (*p* = 0.04) were depleted in the endorhizosphere (Figure 7A).

In particular, four genera (*Pseudomonas*, *Bacillus*, *Streptomyces,* and *Flavobacterium)* alone accounted for about 50% of the bacterial communities of greenhouse-grown plants (Supplementary Figure S7). Horizontal clustering of the heat map for bacteria identified down to the genus level (Supplementary Figure S7) showed that samples from the same compartment (rhizosphere and endorhizosphere and seed endosphere) were clustered on short branches. This thus indicates that the abundances of the rhizosphere samples were similar, as were the endosphere samples, including seeds. Coconut fiber and soil substrates cluster externally.

The differential abundance of fungal communities of plants grown in agricultural soil compared to plants grown in coconut fiber showed that the rhizosphere was enriched in fungi belonging to the genera *Olpidium*, *Plectosphaerella*, *Fusarium*, *Verticillium*, and Hypocreales (all *p* < 0.001) (Figure 7B) and depleted in *Aspergillus, Penicillum* (*p* < 0.001), *Archaeorhizomycetes* (class) (*p* < 0.001), *Rhodotorula* (*p* < 0.001), *Talaromyces* (*p* < 0.001), *Exophiala* (*p* < 0.001), *Pseudeurotium*, *Archaeorhizomyces* (*p* < 0.001), Sordariales (order) (*p* = 0.001), and Pyronemataceae (family) (*p* < 0.001) (Figure 7B). In the endorhizosphere, the taxa Mortierella, *Fusarium*, Helotiales, and *Aspergillus* were enriched, whereas *Olpidium*, *Penicillium*, *Verticillium*, and Talaromyces were depleted (Figure 7B).

Concerning the fungal communities, the horizontal clustering of the samples revealed rather short distances between all the compartments and the substrates (Supplementary Figure S8), except for the rhizospheres in the plants grown in peat (Plant_T1_Rhizo) and in coconut fiber (Plant_T2_CF_Rhizo).

#### 2.2.4. Network Analysis and Keystone Taxa Associated with Bacterial and Fungal Communities

**Figure 8:**
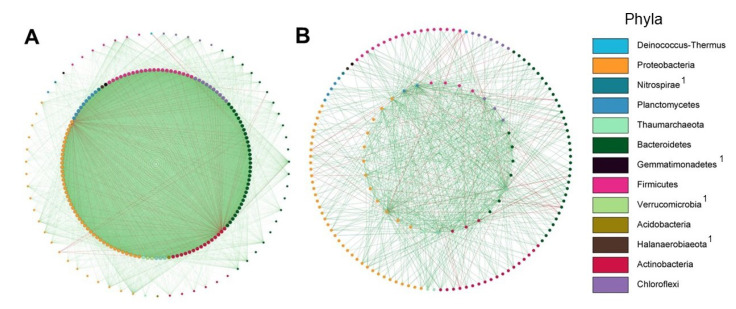
Co-occurrence network analysis: keystone bacterial taxa of the root environment of tomato plants grown in agricultural soil (**A**) and in coconut fiber substrate (**B**). The nodes represent OTUs and the edges represent correlations between and within the keystone taxa nodes and the peripherical taxa nodes. Keystone taxa nodes are depicted as internal to the external peripherical nodes. A connection indicates a statistically significant correlation (FDR adjusted *p* value < 0.05) with positive correlation values >0.6 (green edges) or <−0.6 (red edges). Each node color represents an OTU at phylum level. ^1^ Phyla present only in the soil system as keystone taxa.

Co-occurrence networks of both bacterial and fungal communities from the tomato root environment of plants grown in soil and coconut fiber (soilless) included the rhizosphere and the endorhizosphere data. The network for plants grown in soil consisted of 224 nodes and 14,081 edges (positive 13,828; negative 253) (Table 1). Those in coconut fiber consisted of 217 nodes and 4472 edges (3904 positive; 568 negative) (Table 1). The clustering coefficient was higher in the soilless network (0.41) than in the soil cultivation (0.29). A total of 139 and 33 keystone taxa were detected in soil and soilless conditions, respectively. The keystone OTUs shared between the two networks (Supplementary Tables S18 and S19) belonged to *Proteobacteria, Actinobacteria, Acidobacteria, Bacteroidetes, Chloroflexi, Firmicutes, Planctomycetes*, and *Thaumarcheota* phyla (Figure 8A,B). The OTUs exclusively present in the soil cultivation were assigned to the following orders: Propionibacteriales (*Actinobacteria*), Chloflexales (*Chloroflexi*), Rhodobacterales and Steroidobacterales (*Proteobacteria*) and Opitutales (*Verrucomicrobia*), whereas those exclusively present in the coconut fiber cultivation were in Nitrosphaerales (*Thaumarcheota*), Betaproteobacteriales, and Xanthomonadales (*Proteobacteria*).

In soil and soilless cultivations, 6 and 17 keystone taxa were detected, respectively (Supplementary Tables S20 and S21). Both networks showed OTUs that belong to the *Ascomycota* and *Mortierellomycota* phyla. Unique keystone taxa in tomatoes in the two growing conditions were OTUs in the Hypocreales (*Ascomycota*) order in the soil and Capnodiales, Helotiales, Archaeorhizomycetales and Chaetothyriales (*Ascomycota*), Sporidiobolales, Cystobasidiales and Trichosporonales (*Basidiomycota*) in the coconut fiber.

## 3. Discussion

The rhizosphere microbiome plays an important role in suppressing soil-borne plant pathogens, thereby enhancing the natural suppressive capacity of the soil [11]. In fact, the rhizosphere is home to several species with beneficial traits that affect plant development and plant health and that counteract deleterious soil-borne plant pathogens [28]. The control of soil-borne pathogens in vegetables grown in greenhouses is particularly problematic because it has to incorporate the exclusion or reduction of pathogen inoculum by physical, chemical and biological means, the use of resistant varieties or grafting on resistant rootstocks and important cultivation practices [3,46]. Of these practices, soilless cultivation has several advantages under intensive greenhouse cultivation where a high level of soil-borne pathogens accumulates over time. Ideally, soilless cultivation could lead to a completely pathogen-free production cycle [47].

As soil has been shown to play a more important role than plant genotype on plant and microbiota diversity [30,44], we investigated whether and how the microbiome of the tomato root environment is shaped in plants grown in agricultural soil and in coconut fiber bags, a substrate commonly used in soilless crops.

### 3.1. Tomato Root Microbiome in Soil vs. Soilless Growing Conditions

Consistent with other studies on the bacterial root microbiome, distinct microbial communities were observed in the root compartments of tomato, with a decrease in microbial diversity from the rhizosphere to the endosphere [34,43,48,49,50]. According to the three-step enrichment model of Reinhold-Hurek et al. (2015) [51], for root-associated microbiome assembly from bulk soil to roots, the lowest bacterial richness was observed in endorhizospheres compared to rhizospheres both in the nursery and greenhouse, as well as in the seed endosphere. The fungal communities of the seedling rhizospheres in the peat and in the coconut fiber were characterized by a reduced regularity compared to the other samples. However, these differences were not found for rhizospheres from samples taken from agricultural soils in accordance with other studies [30].

Depending on the substrate/soil in which the tomato plants were grown, the microbial alpha diversity showed the greatest richness and diversity in rhizosphere samples of tomato seedlings that, in the nursery, were grown in peat. In the greenhouse the greatest richness and diversity in rhizosphere samples was observed in plants in agricultural soil, compared to those grown in coconut fiber. A similar result was obtained by Cheng et al., (2020) who observed higher species richness and evenness of bacterial communities in the rhizosphere of tomato plants grown in natural soils than those grown in peat and coconut fiber substrates [44]. The soil composition also affected the microbial community of the rhizosphere and, to a lesser extent, the endorhizosphere [23,33]. Bergna and co-authors (2018) showed that in loamy soil, bacterial communities were characterized by a higher number of rare OTUs and a lower number of dominant OTUs compared to sandy soil [23]. Conversely, the rhizosphere and root endosphere hosted a comparable number of rare OTUs but a lower number of dominant OTUs in plants grown in sandy soil. Taffner et al. (2020) recently observed differences in archaea communities in sandy and loamy bulk soils, while the archaea abundance in the rhizosphere showed a significant soil type-related effect [33].

However, results under commercial conditions are lacking, and our results also showed that the rhizosphere microbiome formed in the nursery is greatly reduced in terms of diversity and richness two months after transplanting into the coconut fiber substrate. The same was not true for plants grown on agricultural land, even though they came from the same batch of nursery seedlings. Nevertheless, the analysis of beta diversity highlights that the soil and the rhizosphere of the plants grown on agricultural land showed clearly different microbial signatures. These signatures were more evident for the bacterial communities.

In each growth environment, bacterial communities again provided important information because the soil or substrate and root compartments in the greenhouse were demarcated; however, they did not differ in the endosphere of seeds and nursery seedlings.

### 3.2. Bacterial Communities Associated with the Root Environment of Tomato

Proteobacteria, Bacteroidetes, and Actinobacteria were the most abundant phyla in the tomato rhizosphere as also observed in other studies [30,52]. However, in some studies, Proteobacteria, Bacteroidetes, and Acidobacteria were found to be the most representative taxa of the rhizosphere of tomato [39,43,52,53]. In our conditions, however, the Acidobacteria phylum represented approximately 2% of the community bacteria in the rhizospheres. The presence of Verrucomicrobia was significant in nursery peat, the seedling rhizosphere and in coconut fiber plants as well as 2% of their respective endorhizopheres, whereas only traces were detected in agricultural soils and in plants grown there. Verrucomicrobia have been found in the rhizosphere of different plant species, and in rice roots, can reach a high percentage both in the rhizosphere and in the endosphere [20,49,54]. Two isolates were cultured and the genomes were sequenced, thus revealing plant growth-promoting traits shared with other soil bacteria [55]. On the other hand, in agricultural soils and the rhizosphere of tomato plants, Chloroflexi was the fourth and fifth phyla represented. Chloroflexi has already been described in the soil and in the rhizosphere of several plant species including tomato [23,30,56,57]. Although reported in numerous microbiome studies, there is little information on Chloroflexi because, together, Acidobacteria and Planctomycetes require more than 12 weeks to grow and form very small colonies on culture media and are therefore very difficult to isolate [58].

Genus-level analysis revealed even greater differences between soil and substrates, rhizospheres and endorhizospheres. The relatively more abundant species, which altogether represented about 50% of bacterial communities in the greenhouse-grown plants, were *Pseudomonas, Bacillus, Streptomyces* and *Flavobacterium.* Relative percentages varied according to the growing conditions (soil or soilless) or compartment (rhizosphere and endorhizosphere). The role of *Pseudomonas*, *Bacillus* and *Streptomyces* as biological control agents and plant growth promoting rhizobacteria (PGPRs) is widely recognized [59,60,61,62,63]. Their action is based on competition for niche and nutrients and direct antimicrobial activity (antibiotics, lipopeptides and enzymes), as well as the induction of systemic resistance [64]. There is much evidence that flavobacteria could also act as PGPR; however, this has not yet been thoroughly examined [65,66,67].

### 3.3. Fungal Communities Associated with the Root Environment of Tomato

Based on phylum-level taxonomy, the fungal communities associated with tomato roots (rhizo and endorhizosphere) were dominated by Ascomycota, Basidiomycota, Olpidiomycota and Morteriellomycota. However, Olpidiomycota, detected in all samples, was the most represented phylum in the rhizosphere of plants grown in agricultural soil. Within this taxon, *Olpidium* was the most represented genus in this phylum. Olpidiomycota dominance was also observed in the endorhizosphere of one of the tomato genotypes in the mycobiome analysis by Manzotti et al. (2020) [32]. *O. brassicae*, the most representative of this species, is a parasite of the epidermis of roots, especially of the Brassicaceae family. It is present in the roots of many major crops and also present in studies of the rhizosphere microbiome [68]. However, Lay et al. (2018) demonstrated that databases confuse *O. brassicae* with the virus carrier *O. virulentus* [68].

In all other samples, Trichocomaceae (Eurotiales, Ascomycota) were strongly represented with the genera *Penicillium* and *Aspergillus* cosmopolitan fungi present in high concentrations in the soils and also in the tomato rhizosphere [69]. The most represented genera in samples of greenhouse grown tomatoes in soil and in coconut fiber were *Plectosphaerella*, *Verticillium*, and *Fusarium*, which were enriched in soil rhizospheres, and fungi in the Sordariomycetes were also enriched. Some of these genera have also been described in a tomato rhizosphere analysis of four genotypes by Manzotti et al. (2020), where the most representative genera were *Plectosphaerella*, *Fusarium*, *Ilyonectria*, *Colletotrichum*, *Pseudeurotium*, *Olpidium*, *Pyrenochaeta* and *Thielaviopsis* [32]. The authors also demonstrated the pathogenic behavior of isolates of *Thielaviopsis, Pyrenochaeta, Fusarium* and *Plectosphaerella* obtained in culture by in planta assays. Zhou et al. (2021) also observed that *Pyrenochaeta* and *Plectosphaerella* were highly enriched in root compartments compared to bulk soil [67]. It is therefore conceivable that fungal pathogenic propagules are present in agricultural soils after repeated cultivations, and since they are attracted by the tomato root exudates, are enriched in the rhizosphere. Under our conditions, no soil-borne diseases were observed that could justify why these taxa are not enriched in the endorhizosphere. In contrast to findings described for bacteria communities, genotype seems to play a major role in the formation of the fungal endophyte communities of tomato roots [23,32].

## 4. Materials and Methods

### 4.1. Experimental Design

Tomato transplants (*Solanum lycopersicum* L. cv “Proxy”) were produced under standard conditions in a nursery in Ragusa, Italy. The seeds were sown in trays filled with blonde sod peat (Klasmann Deilmann Italia S.R.L, Italy). The seedlings were transplanted in a commercial greenhouse located in the same province (36°5103.2400 N 14°27041.4000 E) under two different growing conditions: in agricultural sandy soil typical of the area (sand 70.44%; silt 16.32%; clay 13.24%) or in soilless coconut fiber bags (KOLTURA, Itasmart S.R.L., Italy). Microbiome-formation-related analyses were performed on samples collected at different stages and transferred to the laboratory or to the commercial greenhouse in thermal bags.

Nursery stage T0 and T1: tomato seeds cv Proxy (Seed_T0) and peat substrate (Peat_T0) used for sowing in containers. Root rhizosphere T1 (Plant_T1_Rhizo) and endosphere (Plant_T1_Endo) of tomato plants ready for sale were analyzed. Peat substrate samples were taken directly from three virgin bags with a sterile spoon and transferred to sterile tubes (approximately 50 mL per replicate). Seeds were directly transferred from the producer’s bag into a sterile tube. Tomato transplants (sown on 19 January 2019) were transported in their growing trays to the laboratory for the analysis and to the greenhouse for transplanting (25 February 2019).

Greenhouse growing stage T2: rhizosphere and endorhizosphere of tomato plants at flowering and fruit set after transplanting in agricultural soil (Plant_T2_Soil_Rhizo; Plant_T2_Soil_Endo) and in coconut fiber bags (Plant_T2_CF_Rhizo; Plant_T2_CF _Endo) were sampled. The agricultural soil (Soil_T2) and coconut fiber were tested before transplanting (CF_T2). Samples of agricultural soil and virgin coconut fiber substrate were sampled just before transplanting in the greenhouse. Tomato plantlets were transplanted in two adjacent rows consisting of 20 plants directly into agricultural soil and 20 plants into ten coconut fiber bags (two plants per bag). Plants were drip irrigated. Five bulk samples of the roots from four plants for each condition (soil and soilless) were sampled (23 April 2019).

Five biological replicates were analyzed. Each replica was formed by: 20 seeds, four randomly placed seedlings or plants, and four random samples of substrate and soil samples.

### 4.2. Sample Preparation

To extract the microbial community, each replicate was processed as follows:

- Culture substrates and agricultural soil: 5 g aliquots were suspended in 20 mL of sterile saline buffer (0.85% NaCl) in sterile tubes and vortexed for 1 min.
- Rhizosphere (R): tomato roots were vigorously shaken by hand to remove soil particles. Five grams of roots with firmly attached soil was taken and suspended in 20 mL of saline buffer in sterile tubes and vortexed for 5 min.
- Samples of seeds (S) and root endosphere (E) were collected in sterile tubes with 20 mL of sterile saline buffer. The samples were then washed several times with sterile distilled water. Seeds and root samples were surface sterilized and treated according to the protocol described by Bragina et al. (2012) [70]. Sterility was assessed by placing the surface-sterilized seeds and roots on Potato Dextrose Agar plates (PDA, Oxoid, Milan, Italy) at 27 °C for 4 days. All samples (roots and seeds) were homogenized with a mortar and pestle and suspended in 20 mL of sterile saline buffer.

Four replicates per sample of pellets containing bacteria from both seeds and roots were collected by centrifugation (20 min at 16,750 g) and stored at −80 °C until the DNA extraction.

### 4.3. Metagenomics Analysis

#### 4.3.1. DNA Isolation, PCR Amplification and Sequencing

The pellets were used for total community DNA isolation. DNA was isolated with the FastDNA^®^ SPIN Kit for Soil and the FastPrep^®^ Instrument protocol (MP Biomedicals, Santa Ana, CA, USA) according to the manufacturer’s instructions.

Three technical replicates per sample were subjected to PCR for amplification of the 16S rRNA gene and the fungal ITS region (thermal cycler from Biometra GmbH, Jena, Germany) using Taq-&GO Ready-to-use PCR Mix (MP Biomedicals, Santa Ana, CA, USA) according to Bergna et al. (2018) [23] and Wasserman et al. (2019) [71], respectively. Two different barcoded primers 515f-806r targeting the 16S rDNA hypervariable region 4 [72], and ITS1f-ITS2r to amplify part of the ITS1 * region of the fungal rRNA operon [73]. Barcode sequences for data multiplexing were used as provided by the Earth Microbiome project (earthmicrobiome.org/). Additionally, peptide nucleic acid (PNA) PCR clamps were used to block amplification of plant plastid and mitochondrial 16S rRNA gene sequences during PCR amplification [74]. Technical replicates were combined and purified using the Wizard^®^ SV Gel and PCR Clean-Up System (Promega, Madison, WI, USA), and extracted DNA was quantitated using both the Qubit dsDNA BR Assay Kits (Invitrogen, USA) and Nanodrop 2000 (Thermo Scientific, Wilmington, DE, USA). A total of 126 barcoded samples were equimolar pooled and sent for Illumina MiSeq sequencing (Eurofins Genomics Europe Sequencing GmbH, Germany).

#### 4.3.2. Data Analysis of 16S rDNA and ITS Amplicon for Determination of Microbial Community Structure

Analysis of microbial amplicon data was performed according to Bergna et al. (2018) and Wasserman et al. (2019) [23,71]. The 16S rRNA gene and ITS region sequences were subjected to an initial quality control. Preparation and analysis of raw sequence data were performed using QIIME 2 (Quantitative Insights into Microbial Ecology, version 2019.10) [72,75]. Demultiplexing followed by quality filtering with default QIIME 2 settings was performed for the dataset. High-quality reads were de-replicated and clustered with a 97% similarity threshold via vsearch (v. 2.7.1). After creating a set of representative sequences, the chimeras were filtered through the two approaches based on de novo references while mapping high-quality sequences (vsearch) [76].

Taxonomic assignment was obtained using the QIIME 2 RDP environment (default settings) in combination with the SILVA 16S database (v. 138) [77] for bacterial 16S rRNA and BLAST in combination with the UNITE ITS database for the ITS fungal region (v. 8.1) [78]. Unassigned OTUs and non-bacterial contaminants were filtered from the resulting OTU table. OTU abundances were rarefied via subsampling in the QIIME 2 environment to allow comparisons between samples. A consensus table was obtained by averaging the undersampled tables. The bacterial and fungal community structure was described using a QIIME 2 summary table at the phylum and genus levels with samples belonging to the same microhabitat merged together [77].

Alpha diversity was calculated and rendered at the OTU level in R software (R Core Team, 2013) [79] with the Phyloseq package (v. 1.36.0) [80] using Chao1 and Shannon indices. The PCoA plot was also generated with Phyloseq on OTU tables generated with QIIME 2. Selected OTUs were investigated at greater taxonomically resolved levels with the online nucleotide BLAST tool (blast.ncbi.nlm.nih.gov). Differential abundance analysis was performed with edgeR [81] and limma packages [82] in order to detect enriched and depleted bacterial and fungal genera between samples from the same compartment; statistical analysis was performed with the limma package, taking into account the adjusted FDR *p* *-values.* Data visualization for alpha and beta diversity, correlation and differential abundance analyses were developed with the ggplot2 package [83]. The analysis and visualization of the heatmap were carried out with the ComplexHeatmap R package [84], which was based on the absolute abundance of bacterial and fungal communities in order to estimate, using the Euclidean distance, similarities between samples.

The most abundant bacterial and fungal OTUs of the rhizosphere and endorhizosphere of tomato plants in the greenhouse were used for the construction of co-occurrence networks. Results of the Shapiro–Wilk test showed that the data were not normally distributed; therefore, Spearman’s correlation coefficients (r) were calculated using the Hmisc R package [85]. To highlight the most important interactions, only significant (*p* < 0.05), strongly positive (r > 0.6) and strongly negative (r < −0.6) relationships were taken into account. Cytoscape version 3.9.1 [86] was used for the co-occurrence network visualization. The keystone taxa in agricultural soil and the coconut fiber samples were identified for those bacterial and fungal OTUs with a degree >40, a closeness centrality >0.61 and a betweenness centrality <0.18 according to Berry and Widder [87].

## 5. Conclusions

The present study provides a holistic perspective of the composition, diversity and influential factors shaping the rhizospheric, endophytic, bacterial and fungal communities from nursery stock to greenhouse-grown tomato plants. The results showed that (*i*) the tomato rhizosphere microbiome was shaped by the substrate or soil in which the plants were grown in both the nursery and greenhouse; (*ii*) although tomato soil and soilless crops were started from the same seedlings, the root microbiome diverged widely under the two growing conditions; (*iii*) agricultural soil signatures rely on the enrichment of biological control bacteria of the genus *Pseudomonas* and *Bacillus* and of potential pathogenic fungi of the genus *Fusarium* and *Verticillium;* and (*iv*) bacterial communities in the endosphere are more influenced by seed microbiome than fungal communities. Overall, our study provides a model of the different shaping of the microbial communities in the two cultivation methods, which could explain differences in the response to the introduction of pathogens or the outbreak of opportunistic pathogens in soilless crops. The results also suggest that the exogenous application of biostimulants and beneficial microorganisms needs to take into account the two models described here in order to establish stable and effective interactions [88].

## Figures and Tables

**Table 1:** Network properties of the bacterial and fungal communities in the root environment (rhizosphere and endorhizosphere) of tomato plants grown in agricultural soil and coconut fiber substrate (CF).

Community	Samples	Nodes	Edges	Positive Edges	Negative Edges	Average Clustering Coefficient
Bacteria	Plant_T2_Soil_Rhizo_Endo	224	14,081	13,828	253	0.29
	Plant_T2_CF_Rhizo_Endo	217	4472	3904	568	0.41
Fungi	Plant_T2_Soil_Rhizo_Endo	307	5988	4929	1059	0.31
	Plant_T2_CF_Rhizo_Endo	484	9955	9771	184	0.34

## Data Availability

The data presented in this study are openly available at NCBI database, BioProject under accession number PRJNA863268 (https://www.ncbi.nlm.nih.gov/bioproject/863268, accessed on 22 July 2022).

## Supplementary Materials

The following supporting information can be downloaded at: https://www.mdpi.com/article/10.3390/ijms23158820/s1.
