# Supplementary material for: Soil and Soilless Tomato Cultivation Promote Different Microbial Communities That Provide New Models for Future Crop Interventions
Source: Int J Mol Sci. 2022 Aug 8;23(15):8820. doi: 10.3390/ijms23158820 (PMC9369415; doi:10.3390/ijms23158820)

International Journal of *Molecular Sciences*

MDPI

Soil and soilless tomato cultivation promote different microbial communities that provide new models for future crop interventions

Alice Anzalone[1], Alexandros Mosca[2], Giulio Dimaria[1], Daniele Nicotra[1], Matilde Tessitori[1], Grete Francesca Privitera[3], Alfredo Pulvirenti[3], Cherubino Leonardi[1], Vittoria Catara[1*]

[1] Department of Agriculture, Food and Environment, University of Catania, Catania, Italy.
[2] Department of Physics and Astronomy University of Catania, Catania, Italy.
[3] Department of Clinical and Experimental Medicine, University of Catania, 95123 Catania, Italy.
* Correspondence: vcatara@unict.it; Tel.: (optional; include country code; if there are multiple corresponding authors, add author initials)

Supplementary Figures

Figure S1: Comparison of taxonomic distribution in the samples of the tomato growing chain. The average relative abundances of bacterial (**A**), and fungal (**B**) at the phylum level are represented according to the samples (only key taxa were included, >1%).

Figure S2: PCoA of bacterial **(A)** and fungal **(C)** communities with nursery production materials as a constraining factor; and PCoA of bacterial **(B)** and fungal **(D)** communities with greenhouse commercial materials as a as a constraining factor. The sample clustering was based on the Bray–Curtis dissimilarity matrix. Each dot in the plot corresponds to a single sample (biological replication).

Figure S3: Volcano plots showing differentially abundant bacteria in rhizosphere . Negative log-transformed false discovery rate (FDR) P-values are plotted on the x-axis and log fold change is plotted on the y-axis. The orange dotted horizontal line represents the log fold change cut-off which divides the significant enriched bacteria (red dots) and the significant depleted bacteria (blue dots) from the non-significant (grey dots). The red vertical line represents the 1.30 negative cut-off log p-value (which corresponds to the FDR 0.05 cut-off).

Figure S4: Volcano plots showing differentially abundant bacteria in endorhizosphere. Negative log-transformed false discovery rate (FDR) P-values are plotted on the x-axis and log fold change is plotted on the y-axis. The orange dotted horizontal line represents the log fold change cut-off which divides the significant enriched bacteria (red dots) and the significant depleted bacteria (blue dots) from the non-significant (grey dots). The red vertical line represents the 1.30 negative cut-off log p-value (which corresponds to the FDR 0.05 cut-off).

Figure S5: Volcano plots showing differentially abundant fungi in rhizosphere. Negative log-transformed false discovery rate (FDR) P-values are plotted on the x-axis and log fold change is plotted on the y-axis. The orange dotted horizontal line represents the log fold change cut-off which divides the significant enriched bacteria (red dots) and the significant depleted bacteria (blue dots) from the non-significant (grey dots). The red vertical line represents the 1.30 negative cut-off log p-value (which corresponds to the FDR 0.05 cut-off).

Figure S6: Volcano plots showing differentially abundant fungi in endorhizosphere. Negative log-transformed false discovery rate (FDR) P-values are plotted on the x-axis and log fold change is plotted on the y-axis. The orange dotted horizontal line represents the log fold change cut-off which divides the significant enriched bacteria (red dots) and the significant depleted bacteria (blue

dots) from the non-significant (grey dots). The red vertical line represents the 1.30 negative cut-off log p-value (which corresponds to the FDR 0.05 cut-off).

Figure S7: Heatmap analysis with the dendrogram based on the Euclidean distance. The columns represent the samples and the rows represent the 101 most abundant bacteria identified at the genus level. A darker color indicates a higher bacterial abundance.

Figure S8: Heatmap analysis with the dendrogram based on the Euclidean distance The columns represent the samples and the rows represent fungi identified at the genus level. The absolute abundance of fungi is presented by color in each cell. A darker color indicates a higher bacterial abundance.

Figure S1: Comparison of taxonomic distribution in the samples of the tomato growing chain. The average relative abundances of bacterial (**A**), and fungal (**B**) at the phylum level are represented according to the samples (only key taxa were included ,>1%).

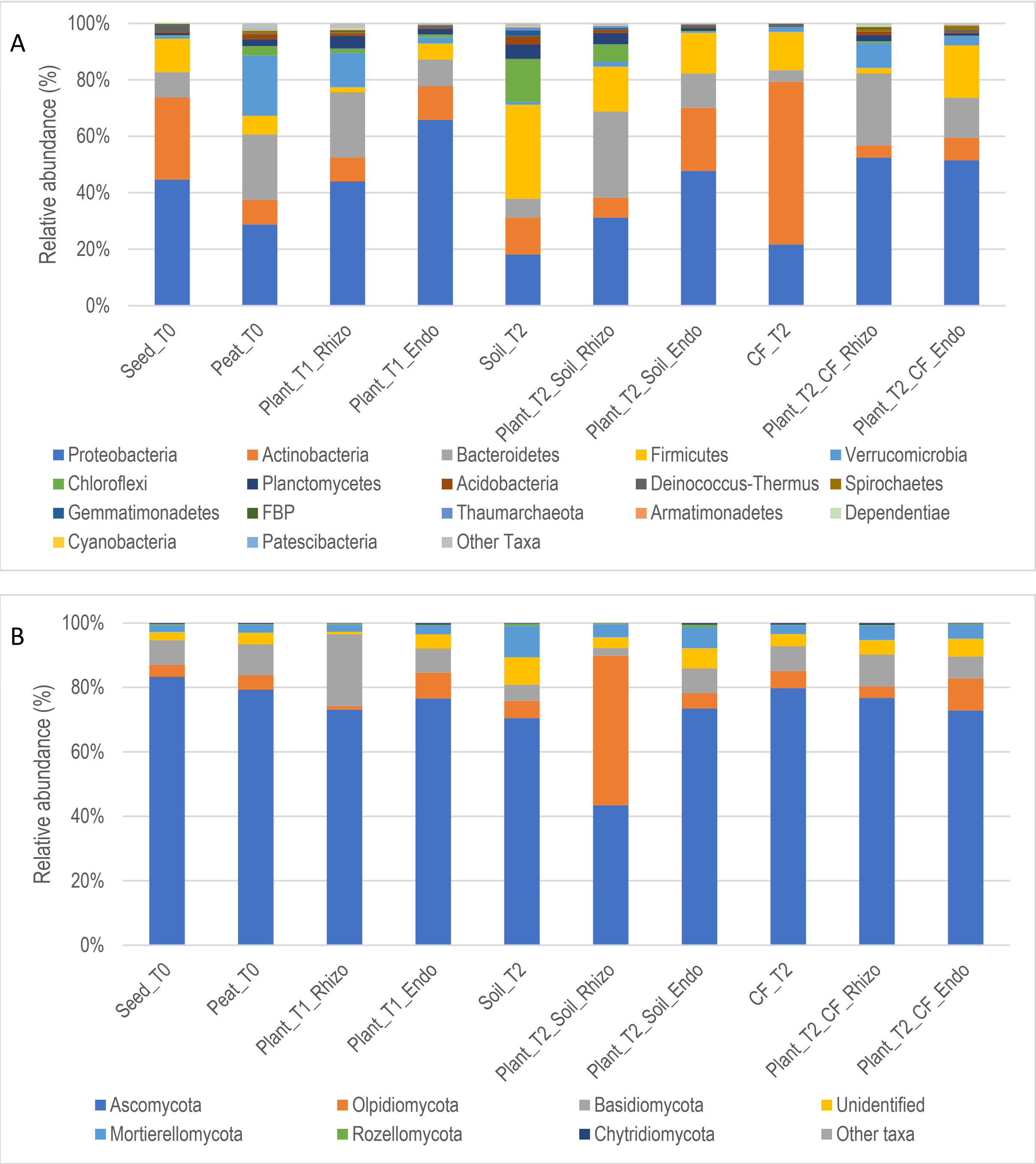

Figure S2: PCoA of bacterial **(A)** and fungal **(D)** communities in the nursery production materials as a constraining factor; and PCoA of bacterial **(B-C)** and fungal **(E-F)** communities with greenhouse commercial materials as a as a constraining factor. The sample clustering was based on the Bray–Curtis dissimilarity matrix. Each dot in the plot corresponds to a single sample (biological replication).

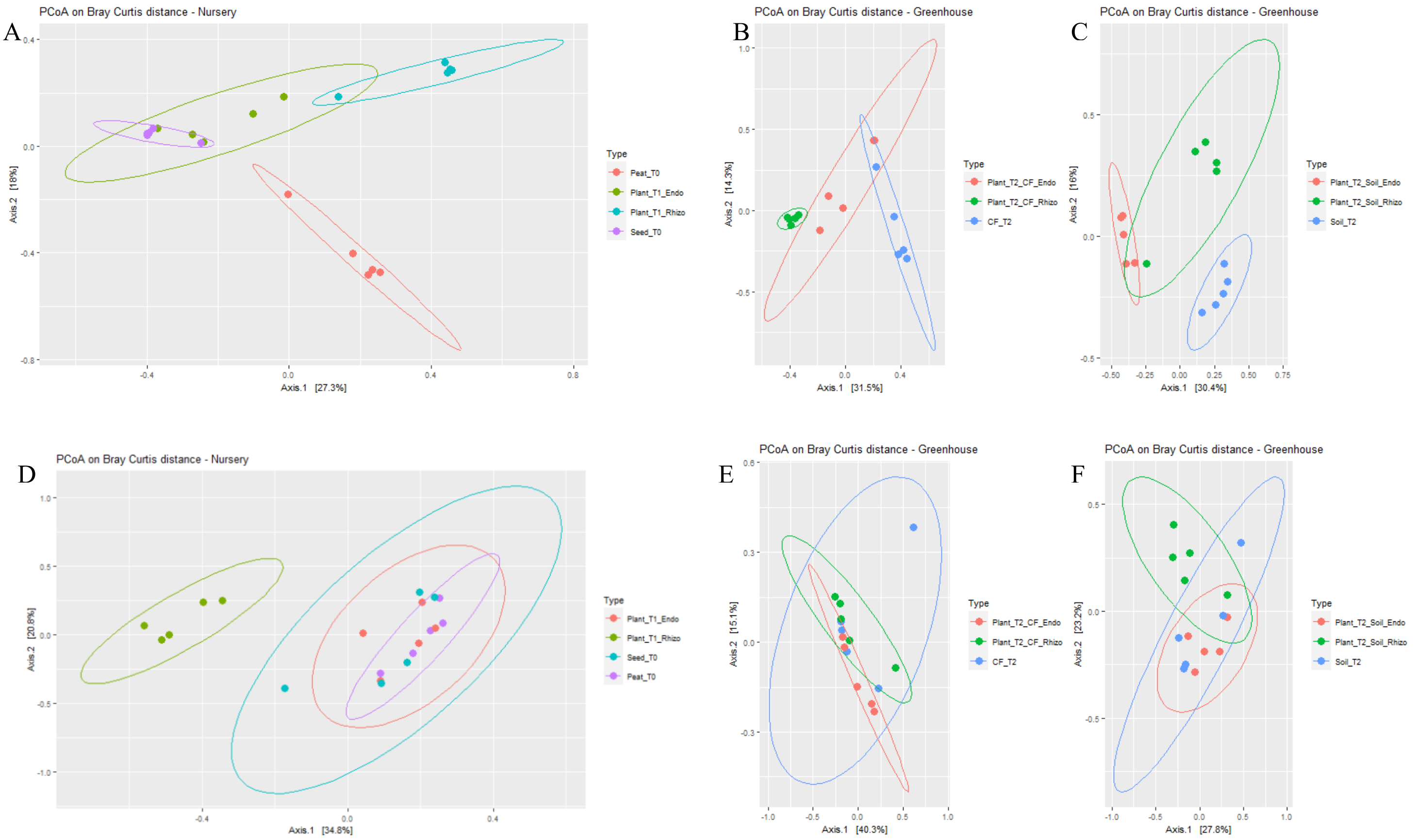

Figure S3: Volcano plots showing differentially abundant bacteria in rhizosphere . Negative log-transformed false discovery rate (FDR) P-values are plotted on the x-axis and log fold change is plotted on the y-axis. The orange dotted horizontal line represents the log fold change cut-off which divides the significant enriched bacteria (red dots) and the significant depleted bacteria (blue dots) from the non-significant (grey dots). The red vertical line represents the 1.30 negative cut-off log p-value (which corresponds to the FDR 0.05 cut-off).

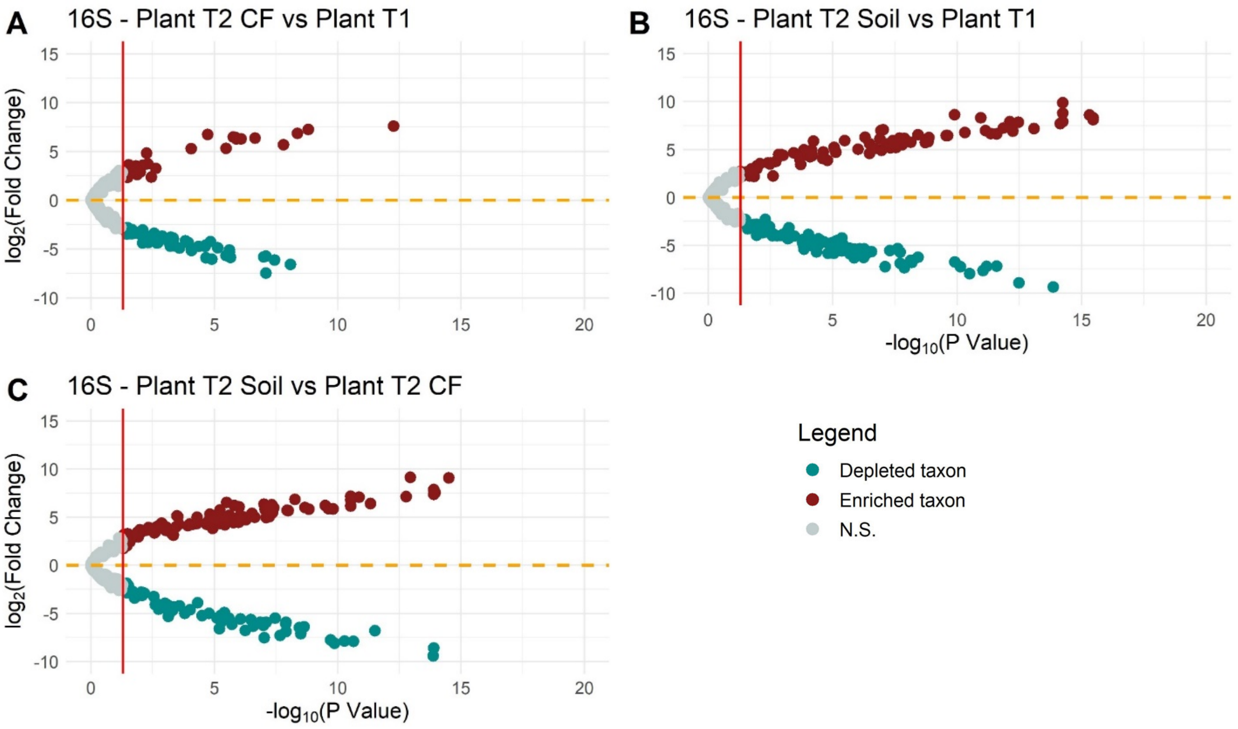

Figure S4: Volcano plots showing differentially abundant bacteria in endorhizosphere. Negative log-transformed false discovery rate (FDR) P-values are plotted on the x-axis and log fold change is plotted on the y-axis. The orange dotted horizontal line represents the log fold change cut-off which divides the significant enriched bacteria (red dots) and the significant depleted bacteria (blue dots) from the non-significant (grey dots). The red vertical line represents the 1.30 negative cut-off log p-value (which corresponds to the FDR 0.05 cut-off).

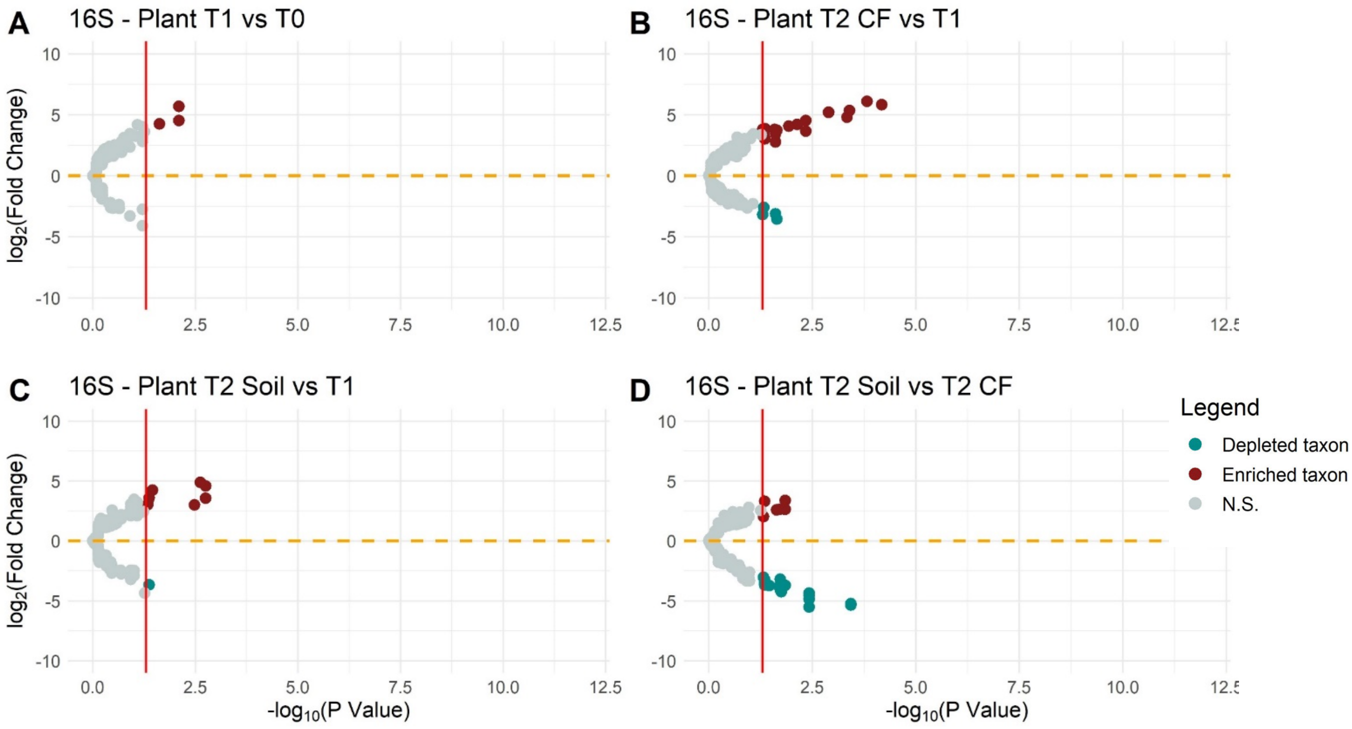

Figure S5: Volcano plots showing differentially abundant fungi in rhizosphere. Negative log-transformed false discovery rate (FDR) P-values are plotted on the x-axis and log fold change is plotted on the y-axis. The orange dotted horizontal line represents the log fold change cut-off which divides the significant enriched bacteria (red dots) and the significant depleted bacteria (blue dots) from the non-significant (grey dots). The red vertical line represents the 1.30 negative cut-off log p-value (which corresponds to the FDR 0.05 cut-off).

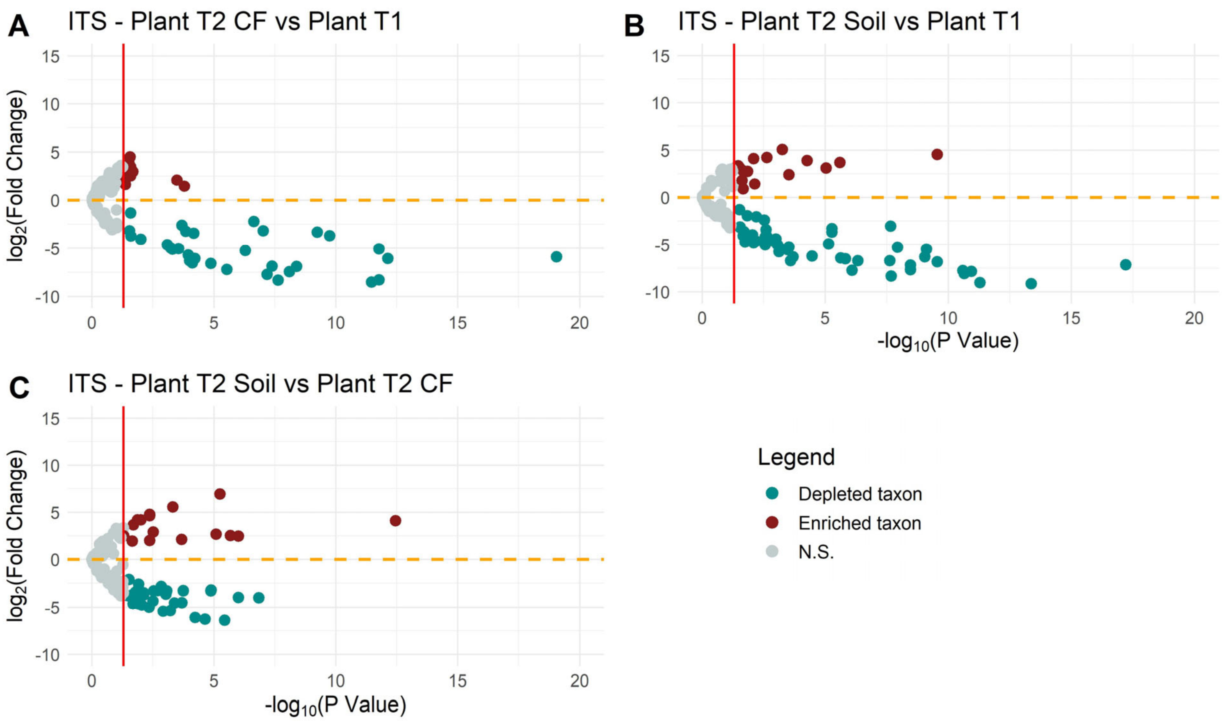

Figure S6: Volcano plots showing differentially abundant fungi in endorhizosphere. Negative log-transformed false discovery rate (FDR) P-values are plotted on the x-axis and log fold change is plotted on the y-axis. The orange dotted horizontal line represents the log fold change cut-off which divides the significant enriched bacteria (red dots) and the significant depleted bacteria (blue dots) from the non-significant (grey dots). The red vertical line represents the 1.30 negative cut-off log p-value (which corresponds to the FDR 0.05 cut-off).

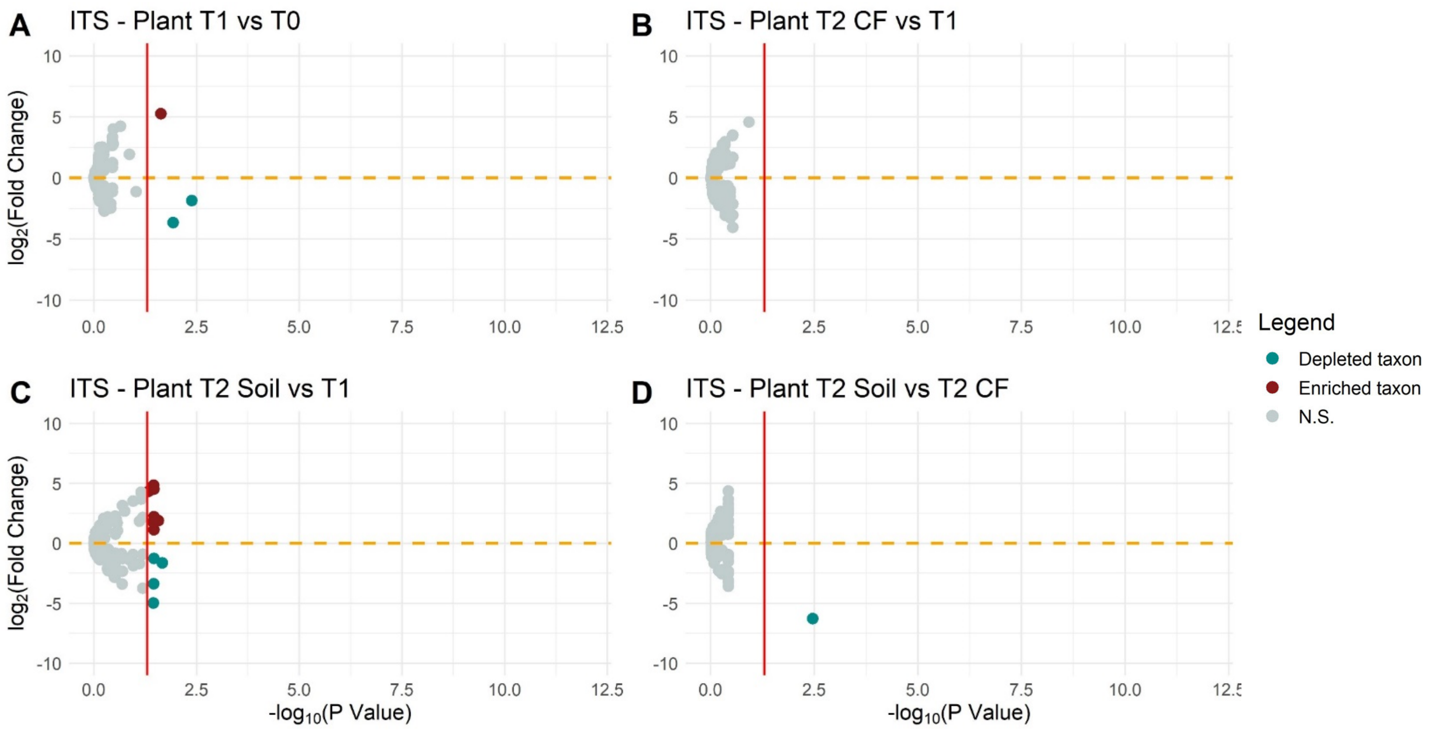

Figure S7: Heatmap analysis with the dendrogram based on the Euclidean distance. The columns represent the samples and the rows represent the 101 most abundant bacteria identified at the genus level. A darker color indicates a higher bacterial abundance.

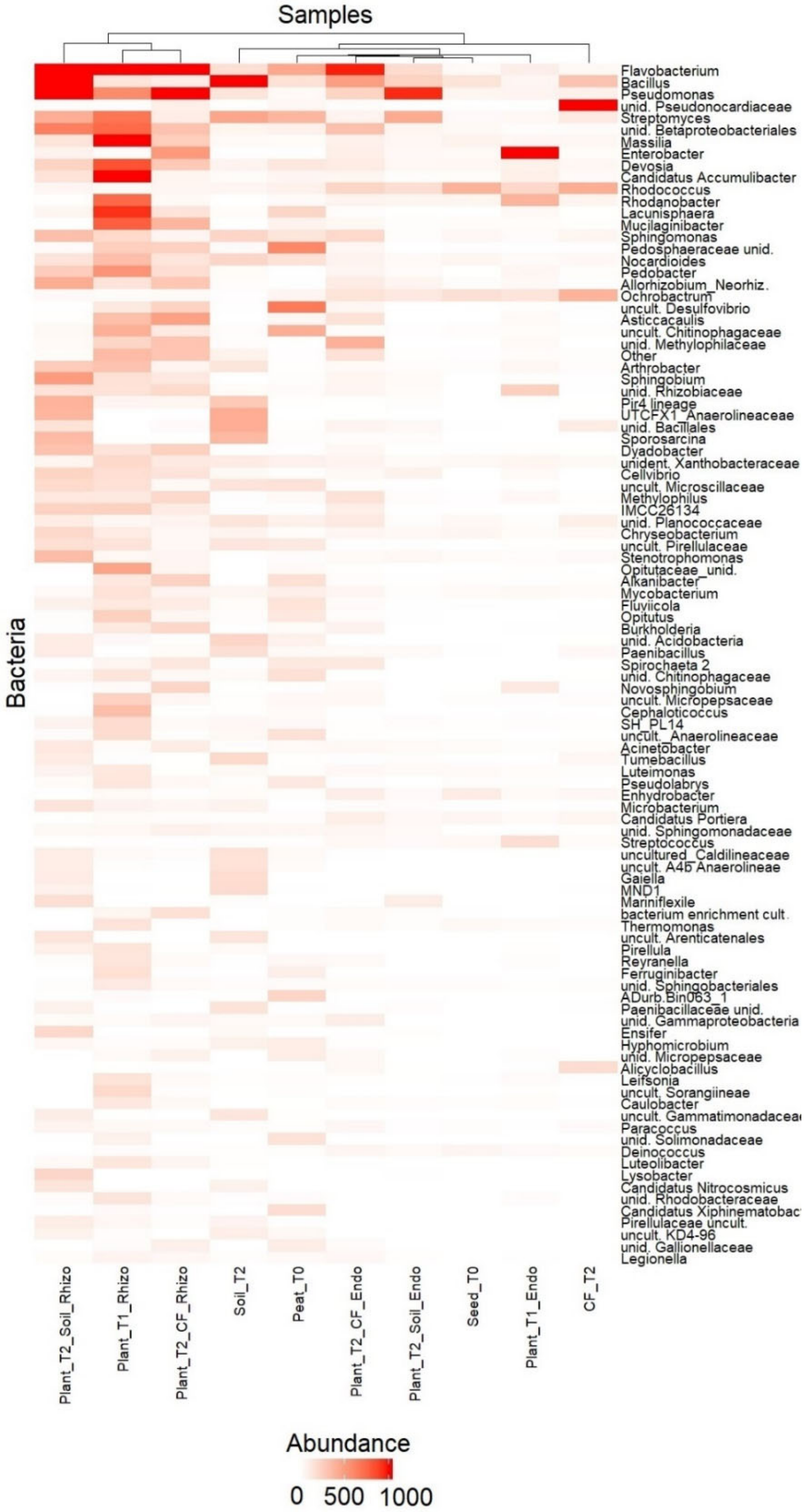

Figure S8: Heatmap analysis with the dendrogram based on the Euclidean distance The columns represent the samples and the rows represent fungi identified at the genus level. The absolute abundance of fungi is presented by color in each cell. A darker color indicates a higher bacterial abundance.

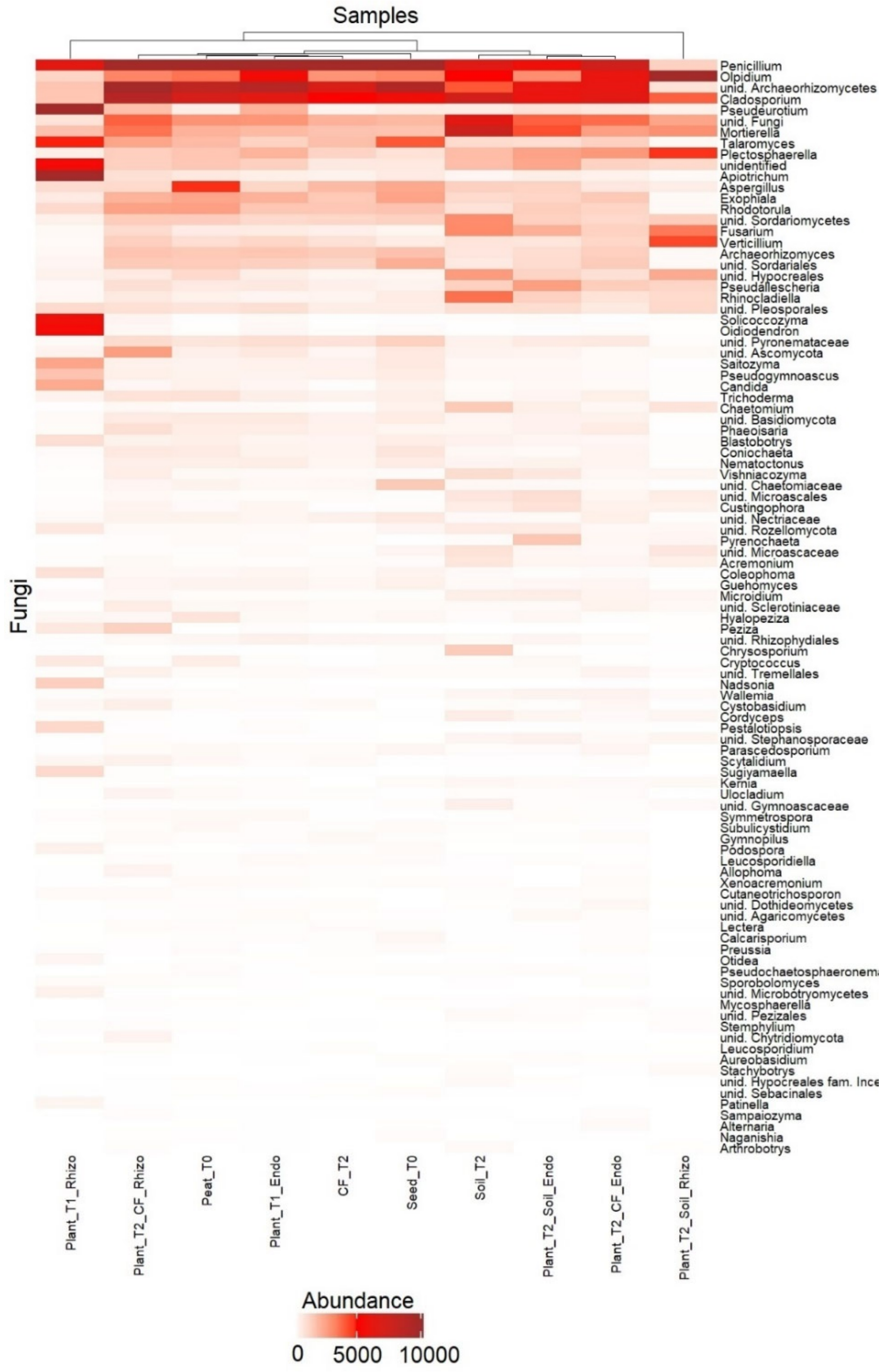

Supplement: Supplementary file 1 [file ijms-23-08820-s001.zip › Supplementary Figures.pdf]
